# Expressive Punctuation and Subjective Adjectives in Biomedical Abstracts for Scholarly Communication

**DOI:** 10.7759/cureus.116515

**Published:** 2026-09-18

**Authors:** Yuki Furuse

**Affiliations:** 1 The University of Tokyo Pandemic Preparedness, Infection and Advanced Research Center (UTOPIA), The University of Tokyo, Tokyo, JPN

**Keywords:** bibliometrics, communication, life science, medicine, publication

## Abstract

Abstracts of academic publications are central instruments of scholarly communication to summarize research findings. In addition, they can signal authors’ stance and evaluation. This study examined how small markers of feeling and stance appear in biomedical abstracts, focusing on expressive punctuation marks and subjective adjectives. We analyzed 23,621,448 PubMed abstracts from journal articles published by 2026, identifying selected expressive punctuation marks, such as “!” and “?”, and subjective participial adjectives, such as “interested” and “surprising”. We then explored their temporal patterns and associations with country of affiliation, authorship, and journal impact factor. We found that these terms were uncommon but far from absent. Question marks appeared in 0.64% of abstracts and exclamation marks in 0.03%. Several markers of curiosity, enthusiasm, and surprise increased over recent decades. Abstracts with East Asian affiliations generally contained fewer such terms, whereas exclamatory usage was most common among abstracts with affiliations in India, and a question mark and subjective adjectives were more common among abstracts with Western affiliations. Abstracts from articles with fewer authors were generally more likely to include expressive punctuation marks and subjective adjectives. Abstracts with inferred female authors, especially inferred female last authors, were more likely to contain “!”, “?”, “interested”, and “surprised”. Conversely, “excited”, “exciting”, “interesting”, and “surprising” were more common in abstracts with inferred male authors. Exclamation marks, question marks, “interested”, and “interesting” were less prevalent in high-impact journals, whereas “shocked” and “surprising” were more prevalent in these journals. These findings suggest that subjective descriptions in scientific writing can reflect broader norms of scholarly communication. Such markers may serve as prompts for considering how abstracts frame novelty, uncertainty, and importance, thereby helping improve scholarly communication.

## Introduction and background

Abstracts of academic publications are not just summaries. They are discovery tools, indexing units, promotional texts, and decision aids for readers, editors, and reviewers. Importantly, scientific writing may not be purely a neutral description. It can also contain small but measurable traces of the authors’ enthusiasm, emotion, or curiosity. Such linguistic features can function as subjective markers, through which authors express stance or evaluation toward their findings. Although often subtle, these markers may influence how readers perceive the novelty, uncertainty, or importance of the reported research.

For example, the use of “surprised” or “interesting” may indicate their subjective evaluation of scientific fact. Such words in scientific writing may be small, but they do considerable rhetorical work. Linguists have long argued that academic writing is not a purely impersonal transfer of facts, but a form of interaction in which authors engage with readers [1]. In fact, studies of spin have shown that wording in abstracts can influence readers’ interpretation of research findings [2-4].

Previous studies have documented increasing promotional or positive evaluative language, such as “novel”, “innovative”, and “promising” in scientific writing [5,6]. Computational approaches have extended this work by applying sentiment-analysis methods, including science-adapted VADER (Valence Aware Dictionary and sEntiment Reasoner) dictionaries and transformer-based classifiers, to large collections of biomedical abstracts [7,8]. Furthermore, gender differences in the presentation of scientific findings have also been reported. In a large analysis of clinical and life-science publications, Lerchenmueller et al. found that publications with male authors were more likely to use positive terms to frame their findings than those with female authors [9].

Although previous studies have examined broad spin or promotional words in scientific writing in biomedical articles, less is known about subjective expressions, such as “?”, “!”, “surprised”, and “interesting”. We therefore investigated the prevalence of subjective adjectives and expressive punctuation marks in abstracts of PubMed-indexed articles, and examined whether their use was associated with bibliometric characteristics, including author and journal profiles.

## Review

Data

All PubMed records were downloaded on June 1, 2026. A total of 40,606,945 records were obtained, of which 31,334,886 were classified as “Journal article”. Reviews, protocols, retraction notices, and other non-original article categories were excluded. Among these, 23,621,448 records contained abstract text and constituted the analytical sample.

For systematic analysis, all PubMed XML (Extensible Markup Language) records were downloaded and processed directly; therefore, no keyword-based PubMed search query was used. For each PubMed article, abstract text, publication year, publication type, author number, author names of the first and last authors, first affiliation of the first and last authors, and journal ISSN (International Standard Serial Number) were extracted from the corresponding XML elements. A code for data retrieval and processing is available on GitHub (https://github.com/yukifuruse1217/emo_pub).

Outcomes

The primary outcome was the use of expressive punctuation marks and subjective adjectives in abstracts. Expressive punctuation marks included exclamation marks, question marks, and their doubled or hybrid forms (“!”, “?”, “!!”, “??”, “!?”, and “?!”). Subjective adjectives were participial pairs as follows: “excited” and “exciting”, “interested” and “interesting”, “shocked” and “shocking”, and “surprised” and “surprising”. For each term, the outcome was its presence or absence in the abstract. These terms were selected because they are visible, easily reproducible markers of stance; we assumed that punctuation marks can signal interrogation or emphasis, and participial adjectives can encode either an authorial reaction or an evaluative property of the finding.

Subjective adjectives were identified using case-insensitive whole-word matching. Expressive punctuation marks were also identified by exact string matching. The search categories were not mutually exclusive; for example, an abstract containing “??” was counted as positive for both “?” and “??”.

Explanatory variables

Countries of affiliation were inferred from the first listed affiliation of the first and last authors using the countrycode package in R; the full affiliation string was evaluated by the algorithm. The names of states in the United States and abbreviations were additionally used to identify U.S. affiliations when an explicit country name was absent. Data from the 20 countries with the highest publication volumes were used in the analysis. For each country, we estimated the proportion of abstracts containing each term of interest separately for first- and last-author affiliations, and then calculated the median of the two proportions.

Author-related explanatory variables included the number of authors and inferred gender of the first and last authors. We focused on the first and last authors because these positions generally represent relatively distinct authorship roles in biomedical publications, with the first author generally representing the lead contributor and the last author often representing the senior or supervisory author [10,11]. The number of authors was grouped into seven categories: 1, 2, 3, 4, 5, 6-10, and ≥11. First- and last-author gender was inferred from first names using the r-gender package in R. Names classified as androgynous or unclassified by the name-based inference algorithm were excluded from the corresponding gender analysis; only names classified as either female or male were included. The terms “inferred female” and “inferred male” are used throughout this article to distinguish algorithmic classification from self-identified gender.

The 2025 journal impact factor, published in 2026, was obtained from Journal Citation Reports by Clarivate and dichotomized as “less than 10” or “10 or higher”. Journal Citation Reports data were linked to PubMed records using journal ISSN identifiers.

Statistical analysis

The prevalence of each term was calculated by year. Temporal trends were examined using Kendall’s rank correlation test between year and the prevalence of each term. A two-sided P-value < 0.05 was considered statistically significant.

Univariable logistic regression models were fitted to estimate unadjusted odds ratios and 95% confidence intervals for the associations of individual bibliometric characteristics with the presence of each linguistic marker. These analyses were intended to describe crude associations rather than independent or causal effects. Associations were considered statistically significant when the 95% confidence interval did not include 1. All analyses were performed in R version 4.5.3. The analysis code is also available on GitHub (https://github.com/yukifuruse1217/emo_pub).

Prevalence of subjective terms

Among the 23,621,448 PubMed records of journal articles with available abstract data, question marks were present in 150,789 abstracts (0.64%), whereas exclamation marks appeared in 6,919 abstracts (0.03%). Doubled or hybrid punctuation marks were even rarer, indicating that highly informal punctuation was uncommon. A statistically significant increase since 1990 was observed for the prevalence of “?”, “??”, “!?”, “exciting”, “excited”, “interested”, and “interesting”, whereas the prevalence of “shocked” declined significantly (Figure 1).

**Figure 1:**
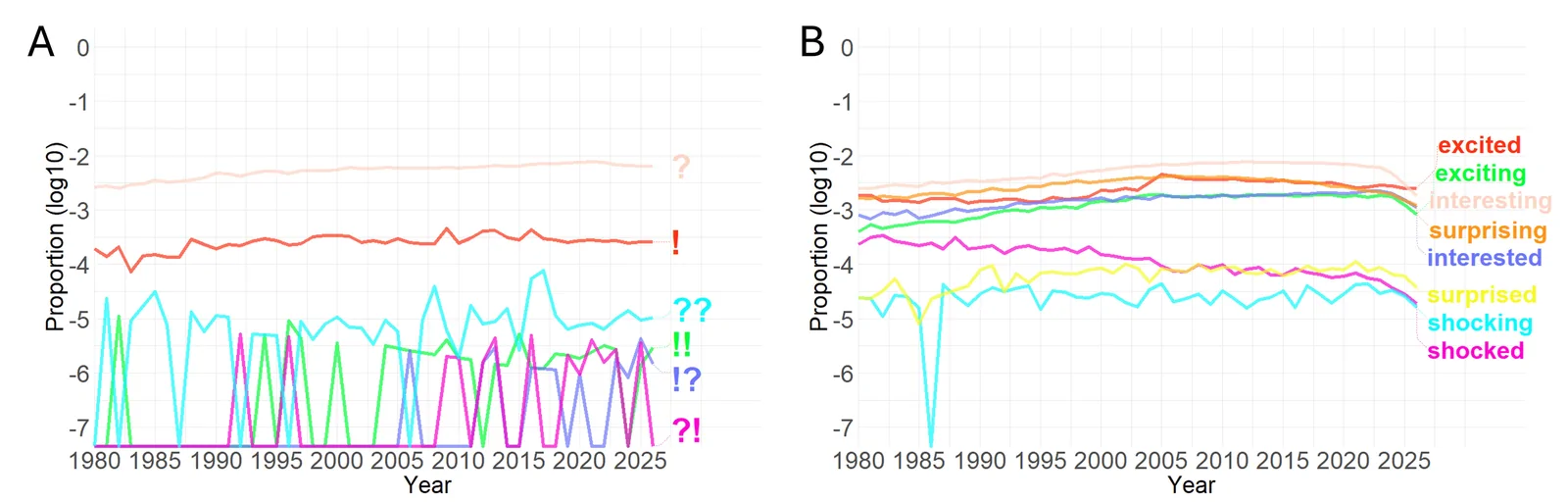
Annual prevalence of subjective terms, 1980-2026 The log10-transformed proportions of abstracts containing each subjective term are shown for expressive punctuation marks (A) and subjective adjectives (B).

The prevalence of these terms across affiliated countries was analyzed for the top 20 countries by publication volume. Table 1 shows the affiliated countries with the highest and lowest prevalence of the investigated terms. Exclamation marks were most strongly associated with affiliations in India, and least with affiliations in China, Japan, and South Korea. Question marks were most common in abstracts from European countries, led by the United Kingdom, and least common in those from China, Japan, and Taiwan. Doubled punctuation marks (“!!” and “??”) were most prevalent in abstracts with affiliations in India, and the hybrid forms (“!?” and “?!”) were observed most frequently in Germany and the Netherlands. Subjective adjectives were generally more prevalent in abstracts with affiliations in Europe and North America and less prevalent in abstracts with affiliations in East Asia. However, “interesting” departed slightly from this pattern, being most frequent in India, followed by Turkey.

**Table 1:** Top five and bottom five countries by prevalence of expressive punctuation marks and subjective adjectives The top 20 publishing countries included in the analysis were AU: Australia; BR: Brazil; CA: Canada; CH: Switzerland; CN: China; DE: Germany; ES: Spain; FR: France; GB: United Kingdom; IN: India; IR: Iran; IT: Italy; JP: Japan; KR: South Korea; NL: Netherlands; PL: Poland; SE: Sweden; TR: Turkey; TW: Taiwan; and US: United States.

Term	Item	1st	2nd	3rd	4th	5th	16th	17th	18th	19th	20th
!	Country	IN	FR	NL	GB	AU	IR	TW	KR	JP	CN
	Prevalence	0.21%	0.13%	0.11%	0.11%	0.11%	0.020%	0.0091%	0.0081%	0.0079%	0.0043%
?	Country	GB	TR	AU	NL	FR	IR	KR	TW	JP	CN
	Prevalence	5.60%	5.10%	5.00%	4.40%	4.20%	1.70%	1.20%	1.10%	0.98%	0.71%
!!	Country	IN	US	TW	ES	GB	-	-	-	-	BR, CA, CH, CN, KR, NL, PL, SE, TR
	Prevalence	0.021%	0.0009%	0.0006%	0.0006%	0.0004%	-	-	-	-	0%
??	Country	IN	TR	TW	PL	US	-	-	-	-	BR, CH, DE, FR, GB, KR, SE
	Prevalence	0.0061%	0.0041%	0.0008%	0.0006%	0.0005%	-	-	-	-	0%
!?	Country	DE	NL	SE	US	BR	-	-	-	-	AU, CN, ES, IR, JP, KR, PL, TR, TW
	Prevalence	0.0012%	0.0010%	0.0005%	0.0004%	0.0003%	-	-	-	-	0%
?!	Country	DE	NL	IR	CH	SE	-	-	-	-	FR, JP, KR, TR, TW
	Prevalence	0.0038%	0.0024%	0.0013%	0.0012%	0.0011%	-	-	-	-	0%
excited	Country	DE	PL	IN	JP	FR	KR	IR	BR	AU	TR
	Prevalence	0.044%	0.042%	0.040%	0.033%	0.032%	0.012%	0.011%	0.011%	0.0086%	0.0048%
exciting	Country	GB	AU	FR	DE	IN	TW	IR	BR	-	KR, TR
	Prevalence	0.0026%	0.0026%	0.0026%	0.0020%	0.0020%	0.0006%	0.0005%	0.0004%	-	0%
interested	Country	AU	SE	GB	US	CA	IN	JP	CN	-	TR, TW
	Prevalence	0.0029%	0.0027%	0.0023%	0.0017%	0.0015%	0.0002%	0.0001%	0.00004%	-	0%
interesting	Country	IN	TR	IT	FR	IR	CN	TW	JP	KR	NL
	Prevalence	0.013%	0.0070%	0.0067%	0.0062%	0.0058%	0.0019%	0.0018%	0.0016%	0.0011%	0.0007%
shocked	Country	FR	AU	IN	JP	SE	ES	-	-	-	BR, IR, KR, TR
	Prevalence	0.0021%	0.0017%	0.0011%	0.0009%	0.0007%	0.0001%	-	-	-	0%
shocking	Country	NL	SE	US	IT	DE	-	-	-	-	AU, CH, ES, IR, JP, KR, PL, TR, TW
	Prevalence	0.0006%	0.0005%	0.0005%	0.0004%	0.0004%	-	-	-	-	0%
surprised	Country	CH	CA	GB	US	DE	-	-	-	-	AU, BR, ES, FR, IN, IR, IT, KR, PL, SE, TR, TW
	Prevalence	0.0015%	0.0005%	0.0004%	0.0003%	0.0002%	-	-	-	-	0%
surprising	Country	DE	CH	CA	US	GB	IN	KR	CN	JP	TW
	Prevalence	0.0059%	0.0056%	0.0053%	0.0048%	0.0042%	0.0012%	0.0007%	0.0006%	0.0005%	0.0002%

Association with author and journal profiles

Articles with fewer authors were more likely to contain expressive punctuation marks and most of the subjective adjectives analyzed (Figure 2). This pattern was weaker for “interesting” and was not apparent for “excited”. “Excited” was observed most frequently in articles with two to five authors.

**Figure 2:**
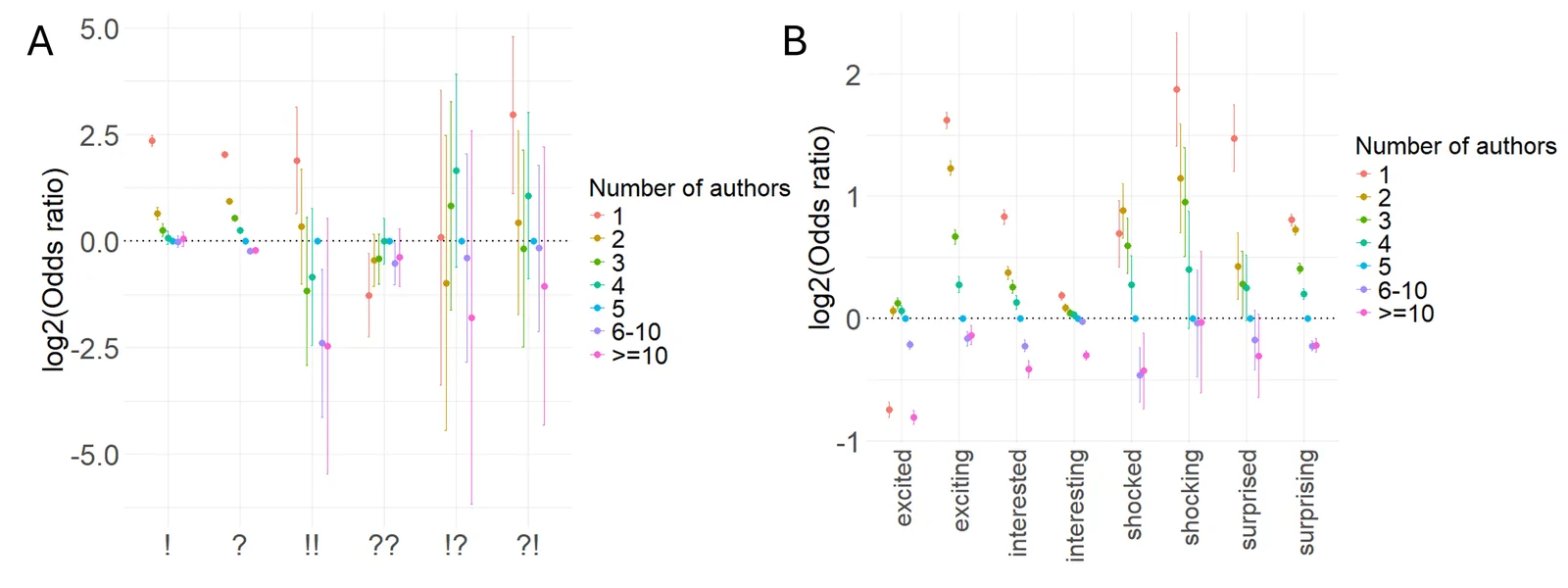
Association between the presence of subjective terms and the number of authors Odds ratios for the association between the presence of subjective terms and the number of authors are shown, with articles with five authors set as the reference group. Panel (A) shows expressive punctuation marks, and panel (B) shows subjective adjectives. Vertical lines indicate 95% confidence intervals.

After excluding names classified as androgynous or unclassified, inferred gender was available for 47.8% of first authors (n = 11,291,013) and 50.1% of last authors (n = 11,833,826); these records constituted the analytical samples for the respective gender analyses. Abstracts with inferred female first or last authors were more likely to contain exclamation marks and question marks, with stronger associations for last authors (Figure 3A). For adjectives, “interested”, “shocking”, and “surprised” were more prevalent in abstracts with inferred female authorship, whereas “excited”, “exciting”, “interesting”, and “surprising” were significantly more prevalent in abstracts with inferred male authorship (Figure 3B).

**Figure 3:**
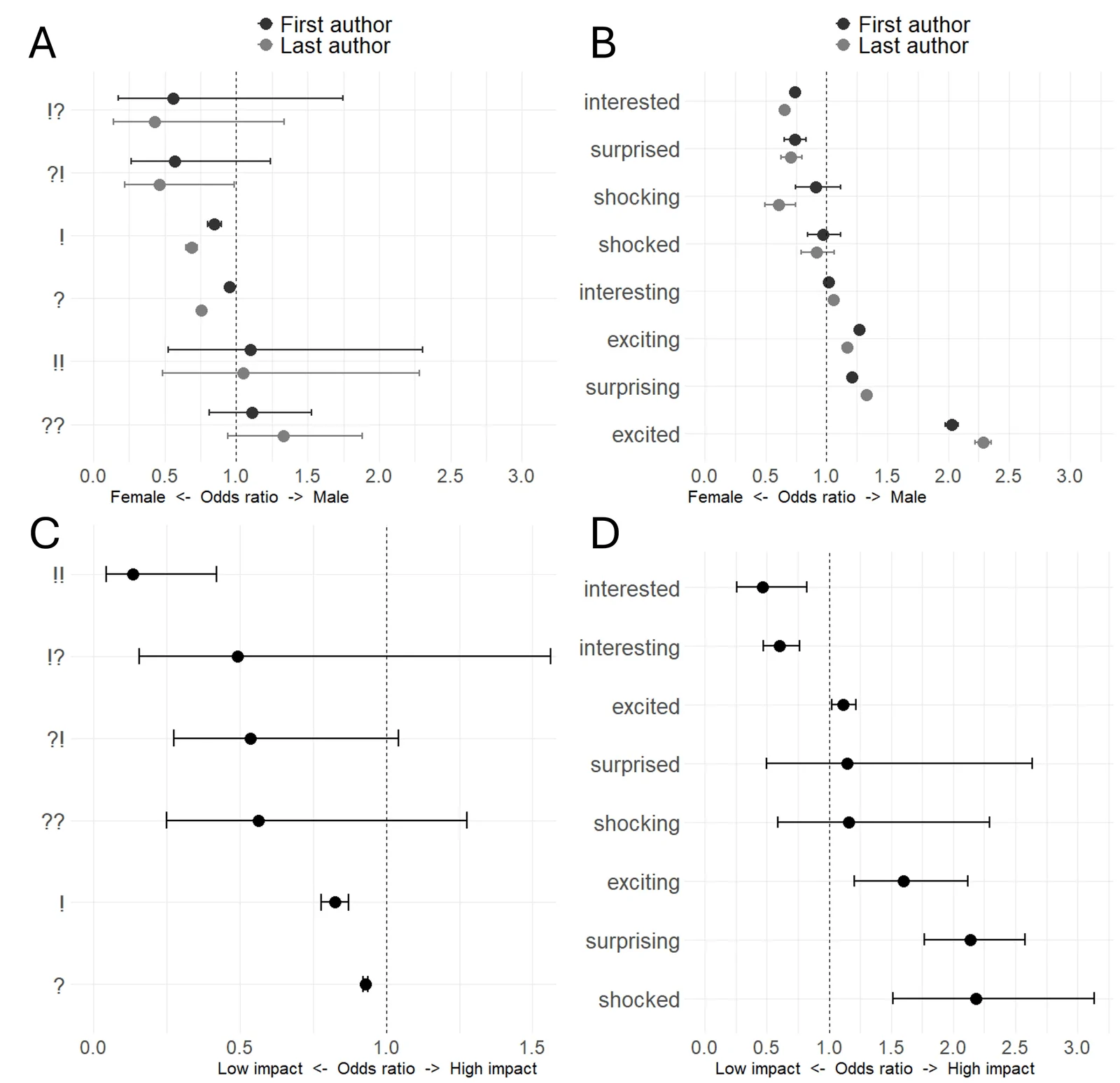
Association between the presence of subjective terms and author gender or journal impact factor category (A, B) Odds ratios for the association between the presence of subjective terms and inferred gender of the first or last author are shown, with female authors set as the reference group. (C, D) Odds ratios for the association between the presence of subjective terms and journal impact factor category (<10 or ≥10) are shown, with lower-impact journals set as the reference group. Variables are ordered by estimated odds ratio. Horizontal lines indicate 95% confidence intervals.

Journal impact factor showed term-specific associations. Exclamation marks and question marks were more prevalent in journals with impact factors below 10 (Figure 3C). “Interested” and “interesting” were also more common in lower-impact journals (Figure 3D). In contrast, “excited”, “exciting”, “shocked”, and “surprising” were significantly more prevalent in high-impact journals.

Discussion

In this analysis of more than 23 million PubMed abstracts, visible markers of stance are uncommon but measurable features of biomedical scientific writing. Question marks appeared in roughly one in 157 abstracts, and exclamation marks in one in 3,413. The distribution of expressive punctuation marks and subjective adjectives across time, authorship, affiliation, and journal characteristics suggests that abstracts are not only summaries of research findings but also products of scholarly communication systems.

Our findings extend previous analyses of scientific language. Previous studies showed that positive and promotional words have increased substantially in biomedical abstracts, suggesting a gradual promotional drift in scientific writing [5,6,9]. Other studies have shown that abstract wording can shape interpretation and may present findings more favorably than the underlying results justify [2-4]. Our study focused on smaller but more playful linguistic features: not broad positivity, but subjective participial adjectives and expressive punctuation marks that more overtly reveal the author’s stance.

We found associations of country and inferred gender with use of subjective terms. There is broader evidence that language use and emotional expression vary across social groups and cultural contexts [12-15]. Yet, the country-level findings in the present study for biomedical abstracts may reflect differences in conventions of academic stance and English-language scientific writing rather than differences in emotional expression per se. Previous studies have demonstrated cross-cultural variation in stance-taking and rhetorical practices in English-language research abstracts, including differences associated with writers’ linguistic backgrounds and translation practices [16,17]. Such variation may arise from multiple mechanisms, including academic writing conventions, English-language proficiency and editing, translation, disciplinary composition, and journal selection.

The study found that inferred female authorship was associated with “!”, “?”, “interested”, and “surprised”, whereas inferred male authorship was associated with “interesting” and “surprising”. Previous large-scale research has identified gender differences in the positive framing of scientific findings, with male authors using promotional terms such as “novel” and “excellent” more frequently [9]. However, the direction of the association in the present study differed among individual lexical forms. The mechanisms underlying these differences cannot be determined from our data and warrant further investigation.

There is a negative association between the number of authors and the frequency of subjective terms. A simple interpretation is that larger author groups act as de facto language review committees; by the time a sufficient number of authors have approved an abstract, someone has removed the subjective wording. High-impact journals showed characteristic patterns for subjective expressions. The positive association of high-impact journals with “surprising” and the negative association with “interesting” may reflect the greater likelihood that important findings are published in those journals. Alternatively, these patterns may partly arise from editorial changes introduced during the publication process.

Limitations

The limitations are equally important. First, our dictionary was intentionally narrow. Many subjective expressions-“remarkable”, “notable”, or “unexpected”-were not assessed in this study. Yet, such positive words have been reported to have increased in biomedical publications [5,6]. Secondly, exact string matching cannot determine the semantic context of individual occurrences [7,18]. We did not apply context-sensitive natural language processing methods, such as transformer-based classifiers, to determine the semantic role of each occurrence. Therefore, some terms identified in abstracts may have been used in technical or other non-subjective contexts; for example, “interested” may describe participants’ interest in a particular subject rather than the authors’ stance, while “shock” may refer to circulatory collapse and “excite” to an electronic state. The identified terms should therefore be interpreted as potential markers of subjective or evaluative stance rather than as direct measures of such stance.

Thirdly, gender was inferred algorithmically from first names. Name-based gender inference is subject to both misclassification and non-classification, and its performance may vary considerably according to linguistic and geographical origin [19]. In particular, inference may be less reliable for non-Western names because naming conventions and the coverage of reference databases differ across populations [20]. The observed associations in this study should therefore be interpreted as associations with algorithmically inferred gender categories rather than with authors’ self-identified gender and may also be affected by selection bias arising from differential classification success.

Finally, the regression analyses were univariable and may therefore be affected by confounding, because bibliometric characteristics such as author number, inferred gender, geographical affiliation, and journal impact factor are interrelated. The reported odds ratios should consequently be interpreted as crude descriptive associations rather than independent or causal effects.

## Conclusions

Expressive punctuation and subjective adjectives are rare but measurable features of biomedical abstracts. Their distribution suggests that stance in scientific writing is shaped not only by authors but also by collaboration, social background, journal prestige, and editorial convention. Subjective descriptions should not necessarily be regarded merely as undesirable stylistic features. Abstracts are used for indexing, searching, screening, and research evaluation. Small linguistic choices may affect how novelty, uncertainty, and importance are perceived; recognizing these effects can help improve scholarly communication among authors, editors, reviewers, and readers.
